# Result of a Kind Action

**Published:** 1860-12

**Authors:** 


### RESULT OF A KIND ACTION.

In September, eighteen hundred and five, a poor young mechanic, just arrived from England, was wandering about New-York in deep dejection ; he was without money, without friends, and without work ; and far from his native home, he knew not which way to turn, but passing along Nassau street, an open door encouraged him to enter. The proprietor was a very little man indeed, perhaps five feet high, but he had a pleasant countenance and a large heart ; for upon being asked by the homeless and penniless stranger if he could direct him to some respectable person who could board him until he could find employment, and thus obtain the means of payment, the storekeeper, pleased with the expression and demeanor of the eighteen-year-old boy, had it in his heart to offer him the desired favor himself ; but he had a wife, whom he knew to be a woman of rare worth, for she was prudent, self-denying, and humane. He might have known what would be her answer, for he had only to make the proposition in a way to indicate his own views, and it would have met with an instantaneous and cheerful acquiescence, unless from some almost insuperable reason. The young stranger was admitted into the family. But the yellow fever was raging in the city. In less than a week the poor lad was stricken with it, and — recovered ! although he was at the point of death for several days. During his illness, he was cared for by his kind host and hostess, with an assiduity and watchfulness which only they know who act from sterling principle and a high humanity. Just a quarter of a century later, this same man was applied to by Major Noah, of pleasant memories, who was then surveyor of the port of New-York, to put together a machine in the Custom-House, and take models of its various parts. This was done, and the mechanic conceived the idea of constructing a similar article, which should excel any thing of the kind for efficiency in the Old World or the New, and he succeeded. He died in eighteen hundred and thirty-three. His son succeeded him in business, and inheriting the inventive genius of his father, combined with rare business tact and indomitable energy, he has added improvement to improvement, until he has made the whole civilized world his debtor. There is not one of all its millions of families which does not every day derive great benefit therefrom. It carries light to every household; hour by hour is lifting the degraded and the fallen, and is aiding in the revolutionizing of all nations which exist by oppression, wrong-doing, and injustice. But that machine, what is it? Fifty years ago one might have been purchased entire for a hundred or two dollars; a common dry-goods box might have easily contained all its parts; but now, in its perfected state, it occupies a space of fifteen feet high and forty feet long; it is made of fourteen thousand seven hundred and thirty parts, weighs fifty thousand pounds, and costs thirty thousand dollars. One of its belongings, not named above, is thirty thousand and sixty-three yards of tape. The penniless English lad was Robert Hoe. The Good Samaritans of Fulton street were Grant Thorburn and his wife, the latter an angel now; the former "still living" in an honored old age, by seven years over four score. The machine is Hoe's ten-cylinder printing-press, as now in operation in the office of the New-York *World*, and the largest ever made.

The first and only newspaper of our childhood was printed on a press which, with the aid of three men, turned out forty or fifty impressions in an hour. When on the twenty-ninth day of November, eighteen hundred and fourteen, the *London Times* announced that it was printed by a machine which made eleven hundred impressions in an hour, the whole city was astonished, and the pressmen themselves looked on in mute wonder and admiration; but to-day, through the agencies of Robert Hoe, the English lad of eighteen hundred and five, of the kindly Grant Thorburn and his wife, and Richard M. Hoe of New-York, there are made at the office of the *World*, in Printing-House Square, twenty-five thousand impressions in sixty minutes. Who can disclaim indebtedness to these four names? The merchant who sips his coffee at breakfast, and reads the latest news up to two or three o'clock in the morning, perhaps forgets to whom he is indebted for that pleasure; and so with the day-laborer, who finds time to glean from his paper, at a cost of one cent, what is going on throughout the habitable globe, ere he sallies forth to his daily toil. Rich and poor, learned and unlearned, all should remember with respect and gratitude the heads and the hearts to which every day makes them renewed debtors, to wit, to Robert Hoe and his son Richard M., to Grant Thorburn and his noble wife.

Reader, remember that kind acts pay; the influence of each for good drifts over the sea of time, and will drift till time shall be no more. Go forthwith, then, "while the day lasts," and perform as many as you can.

